# Relationships that perceived barriers to physical activity have with functional capacity and quality of life in patients with pulmonary hypertension

**DOI:** 10.36416/1806-3756/e20240195

**Published:** 2025-03-18

**Authors:** Layse Nakazato Guedes de Lima, Vitoria Veronez, Paulo Roberto Araújo Mendes, Tatiana Alves Kiyota, Marcos Mello Moreira, Monica Corso Pereira

**Affiliations:** 1. Disciplina de Pneumologia, Departamento de Clínica Médica, Faculdade de Ciências Médicas, Universidade Estadual de Campinas - UNICAMP - Campinas (SP) Brasil.; 2. Programa de Pós-Graduação em Ciências Cirúrgicas, Faculdade de Ciências Médicas, Universidade Estadual de Campinas - UNICAMP - Campinas (SP) Brasil.

**Keywords:** Pulmonary arterial hypertension, Exercise, Motivation, Quality of life, Surveys and questionnaires, Walk test

## Abstract

**Objective:**

Barriers to physical activity can affect the functional capacity and quality of life of patients with pulmonary hypertension (PH). This study aimed to identify the main barriers to physical activity in patients with PH and to examine whether those barriers are related to functional capacity, echocardiographic variables, or quality of life.

**Methods:**

This was a cross-sectional observational study involving 70 patients. Participants scored seven potential barriers to their activities, with a score ≥ 5 indicating a significant barrier. Participants completed the Medical Outcomes Study 36-item Short-Form Health Survey (SF-36) and Manchester Respiratory Activity of Daily Living questionnaire, as well as the six-minute walk test. Correlation analysis, univariate analysis and multiple logistic regression were performed.

**Results:**

As a perceived barrier to physical activity, ‘lack of will’ or ‘lack of energy’ was cited by 67% of the patients. The ‘lack of will’ barrier was found to correlate with all SF-36 domains except bodily pain. We also identified a correlation between the SF-36 vitality domain and the barriers ‘lack of energy’, ‘lack of will’ and ‘lack of structure’. The logistic regression analysis indicated that the vitality domain correlated significantly with the barriers ‘social influence’, ‘lack of energy’, ‘lack of will’, and ‘lack of structure’. For each unit decrease in the vitality score, there was a 10% increase in the probability of citing the barrier ‘lack of will’. No significant correlations were identified between any of the perceived barriers and echocardiographic parameters.

**Conclusions:**

The perceived barrier most commonly reported was ‘lack of will/energy’, which correlated with almost all SF-36 domains, especially vitality. The ‘lack of will’ barrier also correlated with functional capacity.

## INTRODUCTION

Patients with pulmonary hypertension (PH) engage in less physical activity than do individuals with similar demographics.^1^ A variety of tools have been employed to assess reduced physical activity, including accelerometers to count daily steps^2^ ^,^ ^3^; measures of daily energy expenditure and time spent in moderate-intensity activities^3^; and determination of the prevalence of sedentary behaviour.^4^ ^)^ Studies have found that reduced physical activity correlates with self-reported feelings of fatigue^1^ and reduced functional capacity, as measured with the six-minute walk test (6MWT) or one-minute sit-to-stand test.^2^

Reductions in physical activity are primarily attributed to haemodynamic and functional limitations. Psychological factors, such as depression, anxiety and fear of exercise, can contribute significantly to an increase in sedentary behaviour.^5^ However, in clinical practice, health professionals often overlook these aspects for a variety of reasons, including time constraints during consultations, pressure on care processes, unfamiliarity with therapeutic options, uncertainty about the safety of exercise, and a lack of referral services for physical activity.

Cascino et al.^6^ conducted a study on the factors that contribute to low levels of physical activity in patients with PH, despite recommendations.^7^ The study found that health care professionals and patients both lack information on the subject, and questions about the safety and effectiveness of exercise may contribute to the non-referral of patients with PH to exercise programmes. In addition, individuals with PH often face barriers such as low energy levels, lack of motivation and lack of self-discipline, which are associated with low levels of physical activity.^8^

Given the scenario described above, the aim of this study was to identify the main barriers to physical activity in patients with PH and to investigate whether these barriers correlate with demographics or the functional class of dyspnoea. It is also worth investigating whether the barriers identified correlate with physical activity levels, functional capacity, echocardiographic variables, or quality of life.

## METHODS

### Settings

This was a single-centre, cross-sectional observational study conducted between March of 2016 and August of 2021. The sample included in the study was a convenience sample of patients recruited from the PH database of the Pulmonary Vascular Disease Clinic of the Department of Pulmonology of the Hospital de Clínicas, operated by the University of Campinas, in the city of Campinas, Brazil. During the study period, all patients diagnosed with pulmonary arterial hypertension (PAH) or chronic thromboembolic pulmonary hypertension (CTEPH) were screened for eligibility, after which inclusion and exclusion criteria were applied. The study was approved by the Research Ethics Committee of the University of Campinas School of Medical Sciences (Reference nos. 76543617.9.0000.5404 and 28808719.1.0000.5404). All participating patients gave written informed consent.

### Participants

Patients were considered eligible if they were between 18 and 70 years of age, had been diagnosed with PH, classified as World Health Organization group 1 (PAH) or group 4 (CTEPH),^9^ and were stable at New York Heart Association functional class I-III without adjustment to vasodilator therapy for > 60 days. Patients with a diagnosis of depression were excluded, as were those with any cognitive impairment that might make them unable to complete the questionnaires and those with mobility impairments or any muscular or neurological condition that affected their ability to walk or to engage in physical activity. 

### Procedures and variables analysed

Patient records were reviewed to collect clinical data, including those related to demographics, comorbidities and medications. Additionally, echocardiographic data was examined, encompassing measurements of tricuspid regurgitation velocity, systolic pulmonary arterial pressure, tricuspid annular plane systolic excursion, right atrial pressure, right ventricular (RV) strain and S wave velocity.

The six-minute walk test (6MWT) and all three of the questionnaires used were completed on the same day. The instruments employed were the Perceived Barriers to Physical Activity Questionnaire (PBPAQ), the Medical Outcomes Study 36-item Short-Form Health Survey (SF-36), and the Manchester Respiratory Activities of Daily Living questionnaire (MRADL).^10^ ^-^ ^12^

The PBPAQ, created in Brazilian Portuguese,^10^ assesses seven domains considered to be potential barriers to physical activity: ‘lack of time’; ‘social influence’; ‘lack of energy’; ‘lack of will’; ‘fear of injury’; ‘lack of ability’; and ‘lack of structure’. Each domain consists of three questions, each of which is scored on a scale from zero to three, resulting in a domain score range of zero to nine, with a score of five or above indicating a significant barrier. The linear correlation was calculated on the basis of the total scores obtained for each domain. 

The SF-36 is a validated, generic 36-item questionnaire that assesses eight domains: physical functioning; role-physical; bodily pain; general health; vitality; social functioning; role-emotional; and mental health. Scores range from 0 to 100, with lower scores indicating poorer health. The SF-36 has been translated to Portuguese and validated for use in Brazil.^11^

The MRADL is a 21-item scale that measures physical disability and impairment of activities of daily living (ADL) in respiratory disease in four domains: mobility (seven items); kitchen activities (four items); housework (six items); and leisure activities (four items). A perfect total score indicates the absence of any physical disability. The MRADL has also been translated to Portuguese and has been cross-culturally adapted for use in Brazil.^12^

The 6MWT was performed under the supervision of the same technician according to American Thoracic Society guidelines.^13^ The six-minute walk distance (6MWD) was assessed in metres and as a percentage of the predicted value according to a reference equation validated for the Brazilian population.^14^

## STATISTICAL ANALYSIS

To describe the characteristics of the sample, frequency tables were constructed for the categorical variables, showing the absolute and relative frequencies. In addition, descriptive statistics were calculated for the numerical variables, including mean, standard deviation, minimum/maximum values, interquartile range and median. Initial comparisons among groups, stratified by score (≥ 5) for each barrier, to identify potential differences in age, sex, BMI, 6MWD, SF-36 scores and MRADL scores, were performed with the Mann-Whitney test. The chi-square test and Fisher’s exact test were used to test for associations between categorical variables.

Spearman’s correlation coefficient was employed to assess the associations between numerical variables. Univariate and multiple logistic regression analyses were used in order to identify the variables associated with the outcome of interest (a PBPAQ score ≥ 5), including sex, age, functional class, BMI and SF-36 by domain. A stepwise procedure was used in order to select variables. In this process, all possible combinations between variables were examined, with the optimal combination being identified on the basis of the p-value. Bivariate correlations between echocardiographic variables and barriers to physical activity and quality of life were examined by using Pearson’s correlation coefficient for normally distributed data and Spearman’s correlation coefficient for non-normally distributed data.

The significance level for all statistical tests was set at 5%. Analyses were performed using the Statistical Analysis System, version 9.4 for Windows (SAS Institute Inc., Cary, NC, USA), the Jamovi project (2022) software, version 2.3, (https://www.jamovi.org) and the statistical package R, version 4.1 (R Development Core Team, 2021).

## RESULTS

**Figure 1:**
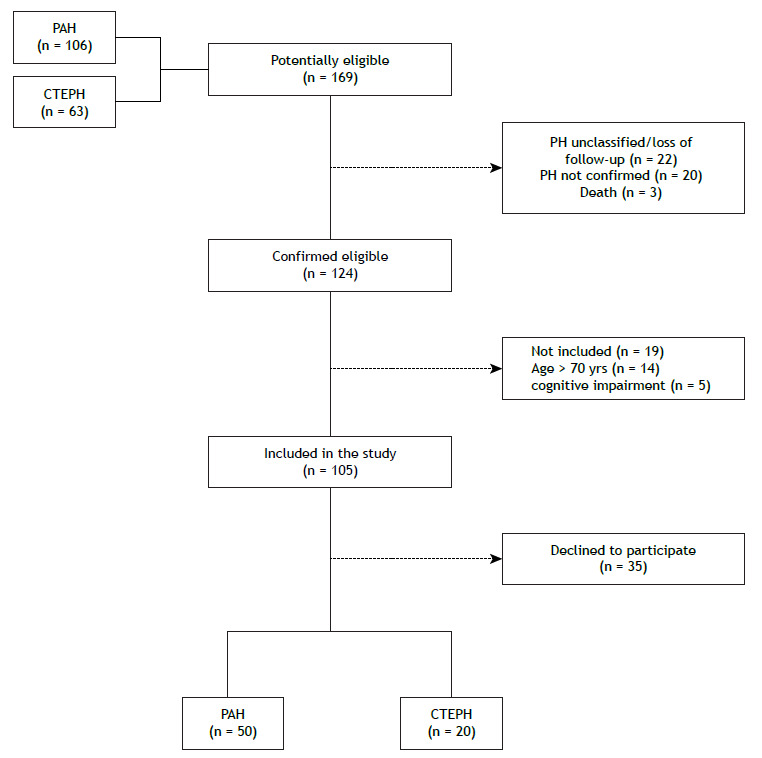
Recruitment flow chart. PAH: pulmonary arterial hypertension; CTEPH: chronic thromboembolic pulmonary hypertension; and PH: pulmonary hypertension.

Of a total of 169 patients recruited, 124 were eligible for inclusion. After the study criteria had been applied, only 70 patients (50 with PAH and 20 with CTEPH) were included and underwent all procedures. Figure 1 shows the recruitment and enrolment process. The mean age of the patients was 44 years, and the majority were women. The baseline demographic, clinical, echocardiographic, and 6MWT data are detailed in Table 1.

**Table 1:** Baseline characteristics of patients with pulmonary hypertension.

Characteristic		(N = 70)
Age (years), mean ± SD		44.0 ± 13.1
Female, n (%)		54 (77.1)
BMI (kg/m^2^), mean ± SD		27.3 ± 6.1
PH classification, n (%)	Idiopathic PAH	30 (42.9)
	PAH associated with connective tissue diseases	11 (15.7)
	PAH associated with congenital heart diseases	9 (12.9)
	CTEPH	20 (28.6)
Functional class (NYHA), n (%)	I	24 (34.3)
	II	31 (44.3)
	III	14 (20.0)
	IV	1 (1.4)
	I + II	55 (78.6)
	III + IV	15 (21.4)
Comorbidities*		46 (65.7)
Echocardiography variables	TRV (m/s)	3.9 ± 0.7
	SPAP (mmHg)	73.4 ± 24.9
	TAPSE (mm)	18.2 ± 3.9
	RAP (mmHg)	9.6 ± 3.6
	RV strain	15.9 ± 4.0
	S wave velocity	10.2± 2.3
Treatment	Monotherapy (sildenafil or ambrisentan or bosentan)	21 (30.0)
	Combination therapy (sildenafil plus ambrisentan or bosentan)	41 (58.6)
	No specific therapy	6 (8.6)
	Use of anticoagulation	42 (60.0)
6MWT^†^	6MWD (m)	
	Mean ± SD	439.00 ± 98.61
	Median (IQR)	447.50 (134.50)
	6MWD (% of predicted^‡^), mean ± SD	73.96 ± 17.60
	6MWD > 440 m, n (%)	34 (55.7)
	SpO_2_ (basal, %), mean ± SD	94.00 ± 3.83
	SpO_2_ (6th min, %), mean ± SD	82.13 ± 17.73
	ΔSpO_2_ (%), mean ± SD	9.38 ± 7.54

PAH: pulmonary arterial hypertension; CTEPH: chronic thromboembolic pulmonary hypertension; NYHA: New York Heart Association; TRV: tricuspid regurgitation velocity; SPAP: systolic pulmonary arterial pressure; TAPSE: tricuspid annular plane systolic excursion; RAP: right atrial pressure; RV: right ventricle; 6MWT: six-minute walk test; and 6MWD: six-minute walk distance. *In both groups-anxiety, arrhythmia, asthma, ascending aortic aneurysm, chronic kidney disease, depression, diabetes mellitus, dyslipidemia, gout, hypothyroidism, obesity, systemic arterial hypertension, rheumatoid arthritis, polyarthritis, portal hypertension, fibrosing interstitial pneumonia, schistosomiasis, thrombocytopenic purpura, and thrombophilia; in the CTEPH group only-mutation in the prothrombin gene, anti-phospholipid syndrome, heterozygous factor V Leiden mutation, and anti-thrombin deficiency. ^†^6MWT: data available for only 68 patients. ^‡^Reference equations for 6MWD taken from Iwama et al.^14^ ^)^

**Figure 2:**
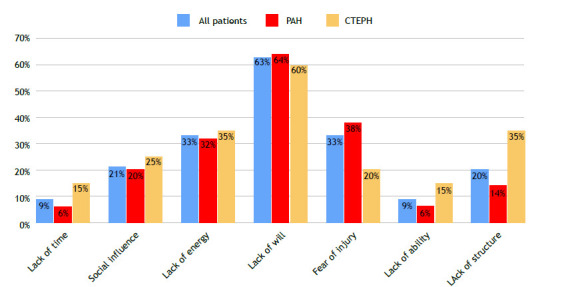
Perceived barriers to physical activity in patients with pulmonary arterial hypertension (PAH) and in patients with chronic thromboembolic pulmonary hypertension (CTEPH).

Using the PBPAQ, we were able to identify the factors most commonly reported by patients as barriers to exercise. ‘Lack of will’ and ‘lack of energy’ were the most common barriers cited by the patients in both groups (Figure 2). Taking into account their semantic similarity and removing overlap (patients who mentioned both reasons at the same time), we found that 67% of all patients mentioned ‘lack of will’, ‘lack of energy’ or both as a barrier to physical activity. 

‘Fear of injury’ was cited by 32.8% of the patients, ‘lack of structure’ was cited by 19.4% and ‘social influence’, which includes a lack of partners or their encouragement to exercise and embarrassment about exercising, was cited by 20.9%. 

Table 2 shows the results of the questionnaires used. In relation to the SF-36 quality of life questionnaire, the domains physical functioning, role-physical and general health were the most affected domains, whereas the role-emotional and mental health domains were moderately affected. Notably, the MRADL questionnaire indicated no significant impairment in the majority of the patients.

**Table 2:** Questionnaire results in patients with pulmonary hypertension (N = 70).

Questionnaire	Subcategory	Median	IQR	Range
SF-36 (score 0-100)*	Physical functioning	45.00	33.75	0-95
	Role-physical	62.50	100.00	0-100
	Bodily pain	62.00	58.75	10-100
	General health	38.50	25.38	0-77
	Vitality	60.00	25.00	10-100
	Social functioning	87.50	50.00	12.5-100
	Role-emotional	83.35	100.00	0-100
	Mental health	58.00	35.00	0-100
PBPAQ (score 0-9)	Lack of time	0.00	2.00	0-9
	Social influence	3.00	3.75	0-8
	Lack of energy	2.00	5.75	0-9
	Lack of will	6.00	3.00	0-9
	Fear of injury	2.00	6.00	0-9
	Lack of ability	0.00	3.00	0-7
	Lack of structure	3.00	1.00	0-9
MRADL (score 0-21)^†^		18.00	5.00	7-21

SF-36: Medical Outcomes Study 36-item Short-Form Health Survey; PBPAQ: Perceived Barriers to Physical Activity Questionnaire; and MRADL: Manchester Respiratory Activities of Daily Living. *A lower score indicates worse health status. ^†^Maximum score = no physical disability.

Table 3 summarises the results of the comparisons between the groups, stratified by barriers with a score ≥ 5, by domain. It is of note that women were more likely to cite ‘lack of energy’ as a barrier to exercising. In addition, older patients cited ‘social influence’ as a significant barrier. It is also noteworthy that patients who more frequently reported ‘lack of will’ as a barrier had more dyspnoea (functional class III or IV) and walked shorter distances on the 6MWT.

**Table 3:** Comparison among groups, stratified by barriers to physical activity with a score ≥ 5, by domain.

Barrier	Variable	Barrier score		p-value
		< 5	≥ 5	
Lack of time (n = 6)	Age (years), mean ± SD	44.41 ± 13.48	39.33 ± 5.99	0.350*
	Female, n (%)	48 (75.0)	6 (100.0)	0.325^‡^
	FC I + II, n (%)	49 (76.6)	6 (100.0)	0.329^‡^
	FC III + IV, n (%)	15 (23.4)	0 (0.0)	
	6MWD (m), mean ± SD	430.77 ± 96.42	464.50 ± 115.75	0.209*
Social influence (n = 15)	Age (years), mean ± SD	42.35 ± 12.90	49.93 ± 12.27	0.023*
	Female, n (%)	43 (78.2)	11 (73.3)	0.734^‡^
	FC I + II, n (%)	45 (81.8)	10 (66.7)	0.286^‡^
	FC III + IV, n (%)	10 (18.2)	5 (33.3)	
	6MWD (m), mean ± SD	442.08 ± 94.22	404.33 ± 107.59	0.321*
Lack of energy (n = 23)	Age (years), mean ± SD	45.17 ± 14.01	41.52 ± 10.73	0.378*
	Female, n (%)	33 (70.2)	21 (91.3)	0.048^†^
	FC I + II, n (%)	39 (83.0)	16 (69.6)	
	FC III + IV, n (%)	8 (17.0)	7 (30.4)	0.226^‡^
	6MWD (m), mean ± SD	441.76 ± 96.20	417.00 ± 101.14	0.478*
Lack of will (n = 44)	Age (years), mean ± SD	46.62 ± 15.73	42.41 ± 11.09	0.293*
	Female, n (%)	19 (73.1)	35 (79.5)	0.534^†^
	FC I + II, n (%)	25 (96.2)	30 (68.2)	0.006^†^
	FC III + IV, n (%)	1 (3.8)	14 (31.8)	
	6MWD (m), mean ± SD	478.46 ± 99.75	406.07 ± 86.60	0.001*
Fear of injury (n = 23)	Age (years), mean ± SD	44.13 ± 13.05	43.65 ± 13.36	0.905*
	Female, n (%)	36 (76.6)	18 (78.3)	0.876^†^
	FC I + II, n (%)	40 (85.1)	15 (65.2)	0.070^‡^
	FC III + IV, n (%)	7 (14.9)	8 (34.8)	
	6MWD (m), mean ± SD	434.83 ± 91.12	431.33 ± 113.63	0.958*
Lack of ability (n = 6)	Age (years), mean ± SD	43.58 ± 13.41	48.17 ± 8.13	0.324*
	Female, n (%)	49 (76.6)	5 (83.3)	1.000^‡^
	FC I + II, n (%)	53 (82.8)	2 (33.3)	0.017^‡^
	FC III + IV, n (%)	11 (17.2)	4 (66.7)	
	6MWD (m), mean ± SD	438.61 ± 97.86	383.50 ± 89.00	0.173*
Lack of structure (n = 14)	Age (years), mean ± SD	42.88 ± 13.43	48.36 ± 10.80	0.140*
	Female, n (%)	43 (76.8)	11 (78.6	1.000^‡^
	FC I + II, n (%)	45 (80.4)	10 (71.4)	0.480^‡^
	FC III + IV, n (%)	11 (19.6)	4 (28.6)	
	6MWD (m), mean ± SD	439.04 ± 96.58	411.38 ± 103.53	0.458*

FC: (New York Heart Association) functional class; and 6MWD: six-minute walk distance. *Mann-Whitney test. ^†^Chi-square test. ^‡^Fisher’s exact test. ^§^Logistic regression modelling of the probability of a barrier score ≥ 5.

The main results of the multivariate regression are presented in Table 4. In summary, the SF-36 vitality domain retained its significance and increased the likelihood of the significant barrier cited being ‘social influence’ (OR = 1.040; 95% CI: 1.006-1.073), ‘lack of energy’ (OR = 1.036; 95% CI: 1.006-1.067), ‘lack of will’ (OR = 1.101; 95% CI: 1.049-1.156) or ‘lack of structure’ (OR = 1.055; 95% CI: 1.016-1.095). The logistic regression analysis confirmed the association between the ‘lack of will’ barrier and the vitality domain of the SF-36, indicating that for each unit decrease in the vitality score, the likelihood of the patient citing ‘lack of will’ as a significant barrier increased by 10.1%. Together, the age and vitality domains of the SF-36 were found to increase the likelihood that the ‘social influence’ barrier would have a significant effect (score ≥ 5): for age, each advancing year increases that likelihood by 6.2%.The full analysis can be found in the supplementary material.

**Table 4:** Logistic regression modelling of the probability that a patient with pulmonary hypertension will give a barrier to physical activity a score ≥ 5.

Barriers with a score ≥ 5	Multivariate logistic regression
Lack of time (n = 6)	Low frequency, test not performed
Social influence (n = 15)	Age and the SF-36 vitality domain together increase the likelihood that a patient would cite social influence as a significant barrier: each advancing year of age increased the likelihood by 6.2%; and each unit decrease in the vitality score increased the likelihood by 4.0%.
	Age: OR = 1.062; 95% CI: 1.008-1.120***
	Vitality: OR = 1.040; 95% CI: 1.006-1.073***
Lack of energy (n = 23)	Vitality was the only SF-36 domain that remained associated with the lack of energy barrier. A decrease of one unit in the score resulted in a 3.6% increase in the likelihood that a patient would cite this as a significant barrier.
	Vitality: OR = 1.036; 95% CI: 1.006-1.067***
Lack of will (n = 44)	Vitality was the only SF-36 domain that remained associated with the lack of will barrier. Each unit decrease in the score resulted in a 10.1% increase in the likelihood that a patient would cite this as a significant barrier.
	Vitality: OR = 1.101; 95% CI: 1.049-1.156*
Fear of injury (n = 23)	Social functioning was the only SF-36 domain that remained associated with the fear of injury barrier. Each unit less in the score increased the chance by 3.8%.
	Social functioning: OR = 1.038; 95% CI: 1.015-1.061*
Lack of ability (n = 6)	Low frequency, test not performed
Lack of structure (n = 14)	Vitality was the only SF-36 domain that remained associated with the lack of structure barrier. Each unit decrease in the vitality score resulted in a 5.5% increase in the likelihood that a patient would cite this as a significant barrier.
	Vitality: OR = 1.055; 95% CI: 1.016-1.095**

SF-36: Medical Outcomes Study 36-item Short-Form Health Survey. *p < 0.001; **p < 0.01; ***p < 0.05.

The Spearman’s correlation coefficient analysis between the SF-36 and PBPAQ scores revealed that the SF-36 vitality domain showed moderate correlations with the barriers ‘lack of energy’ (ρ = −0.395; p = 0.001), ‘lack of will’ (ρ = −0.569; p < 0.0001) and ‘lack of structure’ (ρ = −0.459; p < 0.0001), whereas it showed a weak correlation with the ‘social influence’ barrier (ρ = −0.291; p = 0.015). The barrier ‘lack of will’ showed moderate correlations with all SF-36 domains except bodily pain. The full results of these analyses are presented in the online supplementary material. 

None of the echocardiogram variables were found to correlate significantly with any of the perceived barriers. Pearson’s correlation test revealed that the SF-36 physical functioning domain showed a moderate positive correlation with S wave velocity (R = 0.589; p < 0.001) and with RV strain (R = 0.593). In addition, a moderate positive correlation was observed between the role-physical domain of the SF-36 and RV strain (ρ = 0.603; p < 0.05), whereas a strong positive correlation was observed between the MRADL score and RV strain (ρ = 0.822; p < 0.001). 

## DISCUSSION

Our results show that 67% of the PAH patients identified lack of energy and unwillingness as relevant barriers to exercise. Correlations were found between certain barriers and specific SF-36 domains, vitality in particular. The vitality subscale of the SF-36 measures subjective well-being and consists of four items assessing vitality, energy level and fatigue. 

In clinical practice, patients with PAH often report a ‘lack of energy’ or ‘lack of motivation’ to carry out their ADL. Although these sensations are subjective, their association with quality of life and functional performance demonstrates their clinical relevance. We find it interesting that patients who identified a ‘lack of will as a significant barrier to physical activity have been found to walk shorter distances on the 6MWT, in metres and in percentage of the predicted value.^14^

Fatigue, dyspnoea and chest discomfort on exertion are common symptoms in patients with PAH, with fatigue being reported by 90% of patients.^15^ Fatigue is defined as an intense, persistent feeling of exhaustion that can last for long periods of time. It can significantly reduce the ability to perform ADL, work effectively or engage in other physical activities. It is a multidimensional phenomenon with physical, emotional and cognitive components.^1^ ^,^ ^16^ Fatigued patients experience a lack of energy to engage in physical activity. Cognitively, they report a lack of motivation to engage in their ADL. One study using a multidimensional fatigue inventory to assess fatigue in patients with PH found a high prevalence of intense or very intense fatigue in all dimensions of the inventory: general (60%); physical (55.8%); reduced activity (41.7%); reduced motivation (32.5%); and mental fatigue (27.5%).^17^ Fatigue is associated with a reduction in physical activity and may even lead to a reduction in the overall level of activity. Research suggests that patients with PAH who have lower energy levels also tend to have lower levels of daily physical activity.^1^ ^,^ ^2^ ^,^ ^18^

‘Lack of will’ was the barrier reported by the majority (64%) of the patients in our study sample. That might be related to the ‘lack of interest’ described by Cascino et al.^6^ Those authors investigated perceived barriers to exercise in patients with PAH or CTEPH and used an accelerometer to assess the number of steps taken per day. They found barriers similar to those identified in our study, including ‘lack of self-discipline’, ‘lack of energy’ and ‘lack of interest’. In addition, they found that the barriers ‘lack of interest’, ‘lack of pleasure’ and ‘lack of ability’ were associated with a decrease in daily step counts, a finding also observed by Lima.^19^

In addition to the associations that ‘lack of energy’ and ‘lack of will’ showed with the level of physical activity, we found an association between ‘lack of will’ and the 6MWD. Among patients with PAH, Lima^19^ found an association between the 6MWD and ‘lack of energy and structure’, as well as between the Borg dyspnoea index in the sixth minute and ‘lack of will’. The author also found that several barriers (‘social influence’, ‘lack of energy’, ‘lack of will’ and ‘lack of ability’) were associated with the number of repetitions in the one-minute sit-to-stand test.

In the present study, we found an association between certain barriers and aspects of quality of life, specifically in the SF-36 domains vitality, social functioning, role-emotional and mental health. In patients with PAH or CTEPH, quality of life is impaired because of several aspects of these complex diseases^2^ ^,^ ^19^: their chronicity; the limitations they impose; their impact on the social, professional and socio-economic aspects of the life of the affected patients; the uncertainty of the prognosis; the difficulties in accessing medications and specialised centres; and even the typical delay in receiving a diagnosis of PH.

‘Lack of energy’ and ‘lack of will’ were found to correlate with several SF-36 domains, including those in the mental component (vitality and role-emotional) and those in the physical component (general health, physical functioning and role-physical). The association that the vitality domain showed with both of those barriers was confirmed by multivariate logistic regression. These findings highlight the need for a more qualitative approach to symptoms in the daily care of patients with PAH. Matura et al.^18^ found that the symptoms commonly reported by patients with PAH (dyspnoea on exertion, fatigue and sleep disturbance) were those that most affected their quality of life.

The haemodynamic profile, as assessed by echocardiography, was found to be correlated with the SF-36 (quality of life) domains and with the MRADL score but not with the PBPAQ score. The last was an expected finding. Hemodynamic impairment, assessed here by echocardiography, in measurements already validated with S′ wave and RV strain, affects functional and exercise capacity and thus the level of daily activity. However, it does not necessarily mediate the factors that individuals perceive as barriers to physical activity.

In addition to the current trend toward a more sedentary lifestyle, people who experience fatigue and dyspnoea on exertion are more likely to adopt a less active behaviour, which in turn affects their ability to perform physical activity. It is important to identify potential barriers to exercise and mood disorders. A multidisciplinary approach involving physiotherapists and mental health professionals may help reduce reluctance and increase motivation to exercise. This approach may facilitate the development of strategies that are more comprehensive for the therapeutic management of patients with PH. In addition to haemodynamic impairment, individuals with dyspnoea may have other reasons for not engaging in physical activity, such as fear of adverse events, insecurity about exercising, uncertainty about the need for exercise or its effectiveness, fatigue and lack of energy. Although these subjective aspects are often overlooked in patients with PAH, it is important to consider them.

Our study has some limitations. First, it was a single-centre study with a small sample size and no control arm. However, the fact that the questionnaires employed have been validated in several populations and that their scores are established indicate that our data are valid. In addition, this was a cross-sectional study and causality therefore cannot be established. However, it provides a basis for future research, given that most previous studies on barriers to physical activity in other areas have also been cross-sectional. 

The perception of reduced vitality is common among patients with PH and is a crucial aspect of their well-being and quality of life. Our finding that the SF-36 vitality domain correlated with perceived barriers (‘lack of will’ and ‘lack of energy’), physical activity and functional capacity illustrates the interrelationships among the different aspects assessed here.

Because of the chronic, debilitating nature of PH, its therapeutic management must go beyond medical and surgical treatment. Intrinsic barriers such as ‘lack of energy’, ‘lack of willpower’ and ‘social influence’ are often cited by individuals with PH as reasons for reducing physical activity. Research has shown that these barriers are associated with lower levels of physical activity. A multidisciplinary approach involving physiotherapists and mental health professionals may help reduce reluctance and increase motivation to exercise. Future research aimed at optimising physical activity should include multidimensional interventions that take into account the potential impact of intrinsic barriers. Further studies are needed in order to establish causality between self-reported barriers and the level of physical activity.

## References

[1] Matura LA, Shou H, Fritz JS, Smith KA, Vaidya A, Pinder D (2016). Physical Activity and Symptoms in Pulmonary Arterial Hypertension. Chest.

[2] Nakazato L, Mendes F, Paschoal IA, Oliveira DC, Moreira MM, Pereira MC (2021). Association of daily physical activity with psychosocial aspects and functional capacity in patients with pulmonary arterial hypertension a cross-sectional study. Pulm Circ.

[3] Mainguy V, Provencher S, Maltais F, Malenfant S, Saey D (2011). Assessment of daily life physical activities in pulmonary arterial hypertension. PLoS One.

[4] Pugh ME, Buchowski MS, Robbins IM, Newman JH, Hemnes AR (2012). Physical activity limitation as measured by accelerometry in pulmonary arterial hypertension. Chest.

[5] Chia KSW, Brown K, Kotlyar E, Wong PKK, Faux SG, Shiner CT (2020). 'Tired, afraid, breathless ... .' An international survey of the exercise experience for people living with pulmonary hypertension. Pulm Circ.

[6] Cascino TM, Ashur C, Richardson CR, Jackson EA, McLaughlin VV (2020). Impact of patient characteristics and perceived barriers on referral to exercise rehabilitation among patients with pulmonary hypertension in the United States. Pulm Circ.

[7] Galiè N, Humbert M, Vachiery JL, Gibbs S, Lang I, Torbicki A (2015). 2015 ESC/ERS Guidelines for the diagnosis and treatment of pulmonary hypertension The Joint Task Force for the Diagnosis and Treatment of Pulmonary Hypertension of the European Society of Cardiology (ESC) and the European Respiratory Society (ERS): Endorsed by: Association for European Paediatric and Congenital Cardiology (AEPC), International Society for Heart and Lung Transplantation (ISHLT). Eur Respir J.

[8] Cascino TM, McLaughlin VV, Richardson CR, Behbahani-Nejad N, Moles VM, Visovatti SH (2019). Barriers to physical activity in patients with pulmonary hypertension. Pulm Circ.

[9] Simonneau G, Montani D, Celermajer DS, Denton CP, Gatzoulis MA, Krowka M (2019). Haemodynamic definitions and updated clinical classification of pulmonary hypertension. Eur Respir J.

[10] Amorim PBS (2014). Avaliação objetiva dos hábitos e barreiras da atividade física.

[11] Ciconelli RM, Ferraz MB, Santos W, Meinão I, Quaresma MR (1999). Tradução para língua portuguesa e validação do questionário genérico de avaliação de qualidade de vida SF-36 (Brasil SF-36). Rev Bras Reumatol.

[12] Junkes-Cunha M, Mayer AF, Reis C, Yohannes AM, Maurici R (2016). The Manchester Respiratory Activities of Daily Living questionnaire for use in COPD patients translation into Portuguese and cross-cultural adaptation for use in Brazil. J Bras Pneumol.

[13] ATS Committee on Proficiency Standards for Clinical Pulmonary Function Laboratories (2002). ATS statement guidelines for the six-minute walk test. Am J Respir Crit Care Med.

[14] Iwama AM, Andrade GN, Shima P, Tanni SE, Godoy I, Dourado VZ (2009). The six-minute walk test and body weight-walk distance product in healthy Brazilian subjects. Braz J Med Biol Res.

[15] Matura LA, McDonough A, Carroll DL (2012). Cluster analysis of symptoms in pulmonary arterial hypertension a pilot study. Eur J Cardiovasc Nurs.

[16] Smets EM, Garssen B, Bonke B, De Haes JC (1995). The Multidimensional Fatigue Inventory (MFI) psychometric qualities of an instrument to assess fatigue. J Psychosom Res.

[17] Tartavoulle TM, Karpinski AC, Aubin A, Kluger BM, Distler O, Saketkoo LA (2018). Multidimensional fatigue in pulmonary hypertension prevalence, severity and predictors. ERJ Open Res.

[18] Matura LA, McDonough A, Carroll DL (2016). Symptom Interference Severity and Health-Related Quality of Life in Pulmonary Arterial Hypertension. J Pain Symptom Manage.

[19] Lima LNG (2019). Correlation between physical activity level with functional and quality of life variables in patients with pulmonary arterial hypertension [dissertation].

